# Fracture of the Patella

**Published:** 1872-07-01

**Authors:** W. D. Barclay

**Affiliations:** Muscatine, Iowa


### FRACTURE OF THE PATELLA.

A CASE BY DR. W. D. BARCLAY, MUSCATINE, IOWA.

A gentleman, fifty-five years old, in getting out of a wagon, lost his balance and fell on the ice, making a transverse fracture of the left patella. Three days after I was called, the tumefication and pain was so great, he suspected something wrong. I could indent the tumor sufficient to get my fingers between the pieces of bone, and easily move the pieces in separate directions.

The treatment consisted in a continuation of pouring cold water on the limb for twenty-four hours, reducing the swelling. I took adhesive straps three-quarters of an inch wide (the leg being in a straight position), drawing the lower part of the bone up—the straps running transversely, and up to the lower end of the femur—and the upper half drawn down in the same way, until I had perfect apposition of the bone ; thence transversely over the fractured parts to hold them in position. The splint consisted of flannel cloth (double), reaching from the upper third of the femur to the lower third of the leg ; sewing two seams up the center of the cloth, one inch apart, for the purpose of a hinge ; stitching the upper layer of cloth to the inequalities of the limb, making a perfect fit. Mixing gypsum to the consistency of thick cream; spreading it three-eighths of an inch evenly over the cloth that covers the leg ; then sewing up the outside part evenly before the plaster is set. The splint is then perfect to remove and put on as often as necessity requires.

At the end of six weeks the bone was united, the splint removed, and passive motion given. At eight weeks the adhesive straps were removed, and the patient walked with a crutch, and at twelve weeks walked comfortably, bearing all his weight on his leg. Cured.

Bromo-Chloralum.—This is a very efficient antiseptic, disinfectant, and deodorizer. It possesses the decided advantage of being in itself inodorous and nearly devoid of irritant properties, when locally applied. The writer has used it in many instances as a substitute for solutions of carbolic acid, with very pleasant results. It is not an anæsthetic sedative, like the latter, in its local application, and hence, in many instances, is preferable. We consider it a valuable addition to our medical armamentarium.—*Chicago Med. Jour.*

